# Supplementary material for: Neuroprotective potential of intranasally delivered L-myc immortalized human neural stem cells in female rats after a controlled cortical impact injury
Source: Sci Rep. 2023 Oct 19;13:17874. doi: 10.1038/s41598-023-44426-7 (PMC10587115; doi:10.1038/s41598-023-44426-7)

**Scientific Reports**

**Supplementary Information**

**Neuroprotective potential of intranasally delivered immortalized L-myc (LM-NSC008) human neural stem cells in adult female rats after a controlled cortical impact injury**

Mari Amirbekyan^1^*, Vikram Adhikarla^4*^, Jeffrey P. Cheng^2,3^*, Eleni H. Moschonas^2,3,5^, Corina O. Bondi^2,3,5,6^, Russell C. Rockne^4^, Anthony E. Kline^2,3,5,7-9, #^ and Margarita Gutova^1, #^

^1^Department of Stem Cell Biology and Regenerative Medicine, Beckman Research Institute, City of Hope National Medical Center; 1500 E Duarte Rd, Duarte, CA 91010, USA

^2^Physical Medicine & Rehabilitation, University of Pittsburgh, Pittsburgh, PA

^3^Safar Center for Resuscitation Research, University of Pittsburgh, Pittsburgh, PA.

^4^Department of Computational and Quantitative Medicine, Division of Mathematical Oncology and Computational Systems Biology, Beckman Research Institute, City of Hope National Medical Center; 1500 E Duarte Rd, Duarte, CA 91010, USA

^5^Center for Neuroscience, University of Pittsburgh, Pittsburgh, PA.

^6^Neurobiology, University of Pittsburgh, Pittsburgh, PA

^7^Center for the Neural Basis of Cognition, University of Pittsburgh, Pittsburgh, PA

^8^Critical Care Medicine, University of Pittsburgh, Pittsburgh, PA

^9^Psychology, University of Pittsburgh, Pittsburgh, PA


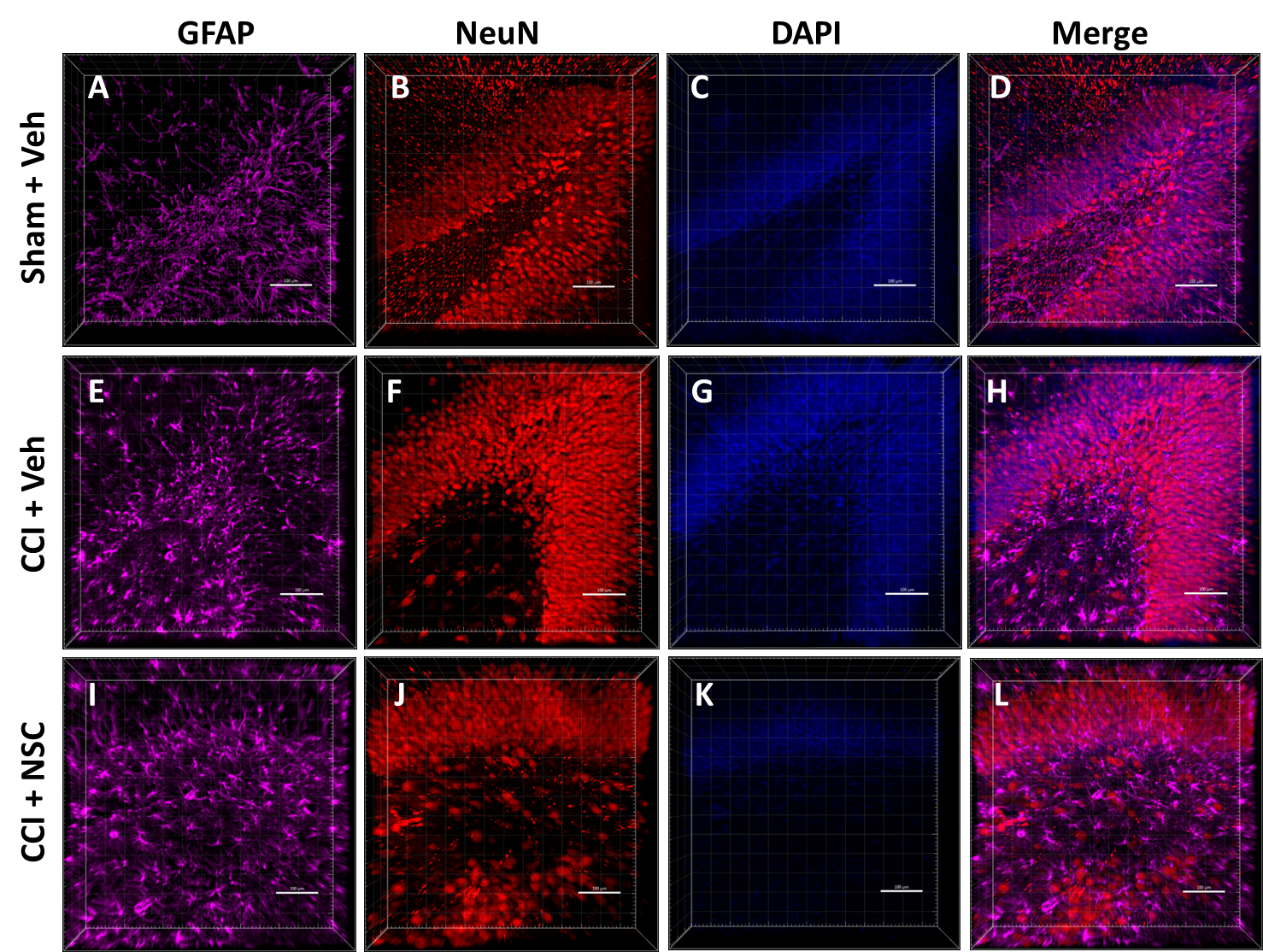


**Figure S1. Lack of NSCs in neurogenic zones (hippocampus) on the contralateral side after intranasal administration to female rat brain.** No eGFP signal is present in any of images analyzed. Staining of astrocytes (far red), maturing neurons (red), and nuclei (blue) was done on 1 mm-thick coronal rat brain sections. 3D images of Z-stacks taken at 10X magnification through 250-350 µm of tissue show individual and merged color channels as well as the localization of eGFP-labeled LMNSC008 cells 39 days post-surgery of three female rat brains from groups: SHAM + VEH, CCI + VEH, and CCI + NSC. See Figure 1 for quantifications of NeuN- and GFAP-stained cells.


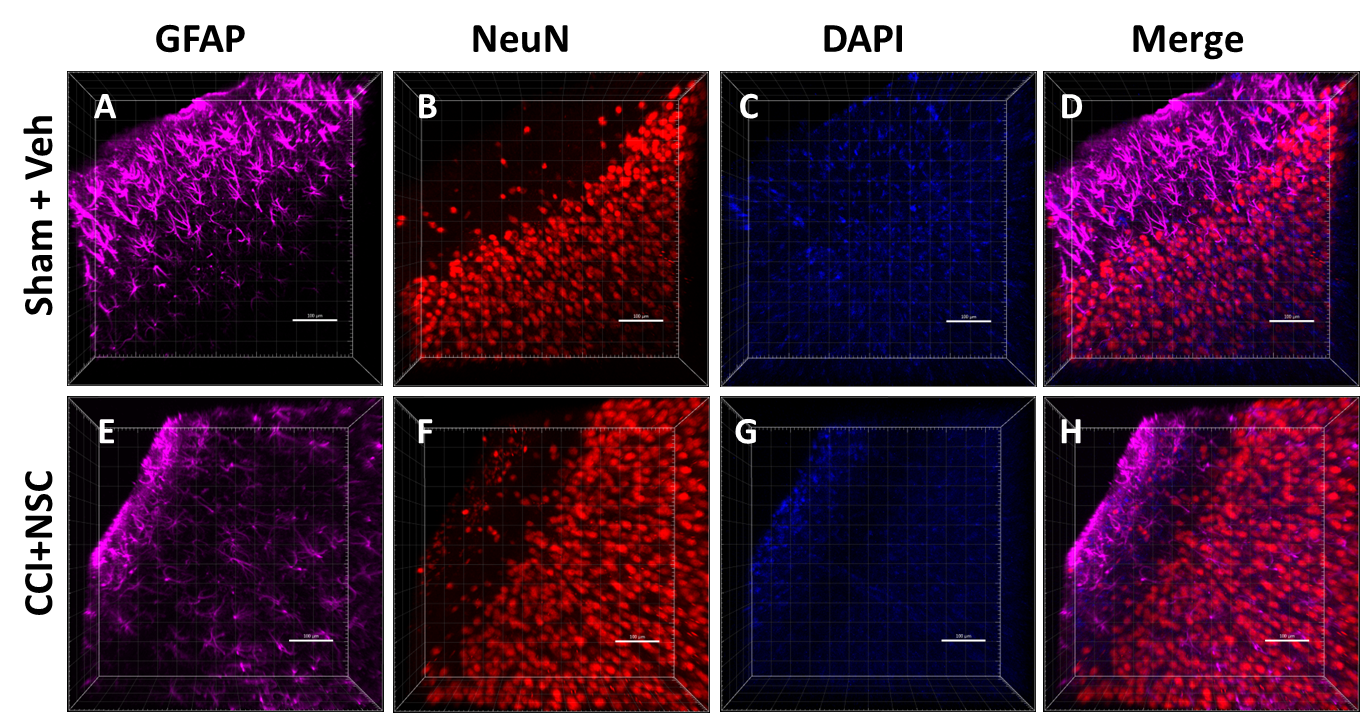


**Figure S2. Lack of NSCs in the cortical areas of the contralateral hemisphere after intranasal administration.** Staining of reactive astrocytes (far red), maturing neurons (red), and nuclei (blue) was done on 1 mm-thick coronal rat brain sections. 3D images of Z-stacks taken at 10X magnification through 250-350 µm show individual and merged color channels as well as the localization of eGFP-labeled LMNSC008 NSCs 39 days post-surgery of female rat brains (n=4) from groups Sham + VEH and CCI + NSC. CCI + VEH section staining unavailable for display. Lack of visible eGFP signal in these areas indicates either limited or absolute restriction of NSC migration to these areas and to the contralateral hemisphere in general.

**
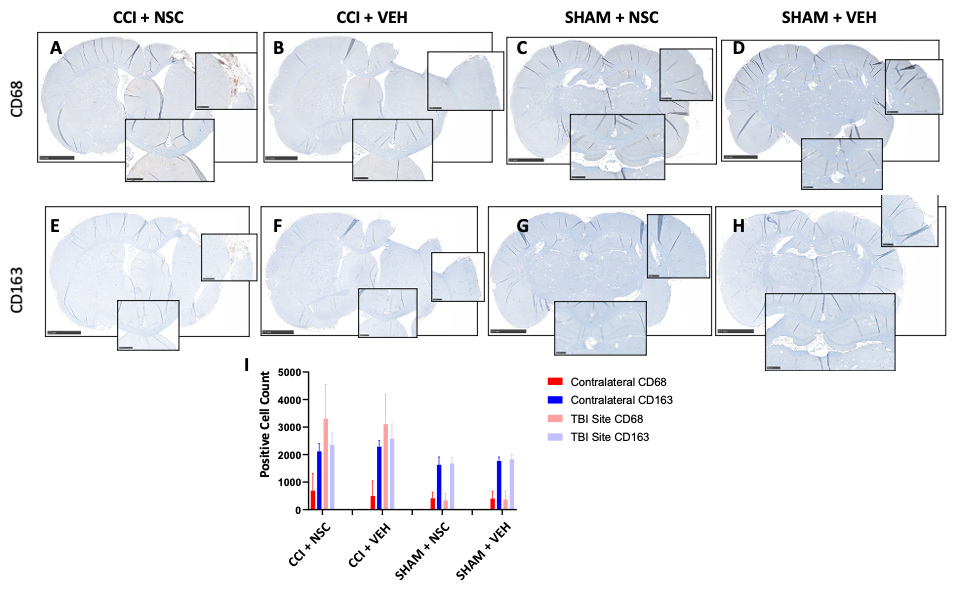
**

**Figure S3. Comparative IHC quantification of contralateral and TBI paraffin-embedded female rat brain M1/M2 myeloid immune cells across all four groups.** (A-D) IHC staining of the TBI hemisphere and contralateral [CL] hemisphere for CD68 expression; (E-H) IHC staining of the TBI and CL hemisphere for CD163. (I) QuPath software was used to separately quantify CD68 and CD163 intensities in the CL or TBI hemisphere of each brain. Five paraffin-embedded brain sections were analyzed per animal and brains from four individual rats were analyzed per group. Results from each group were averaged by mean and +SD. Compared to the SHAM controls, the CCI groups had greater CD68 staining in the TBI hemisphere, while the CL remained the same as the controls.


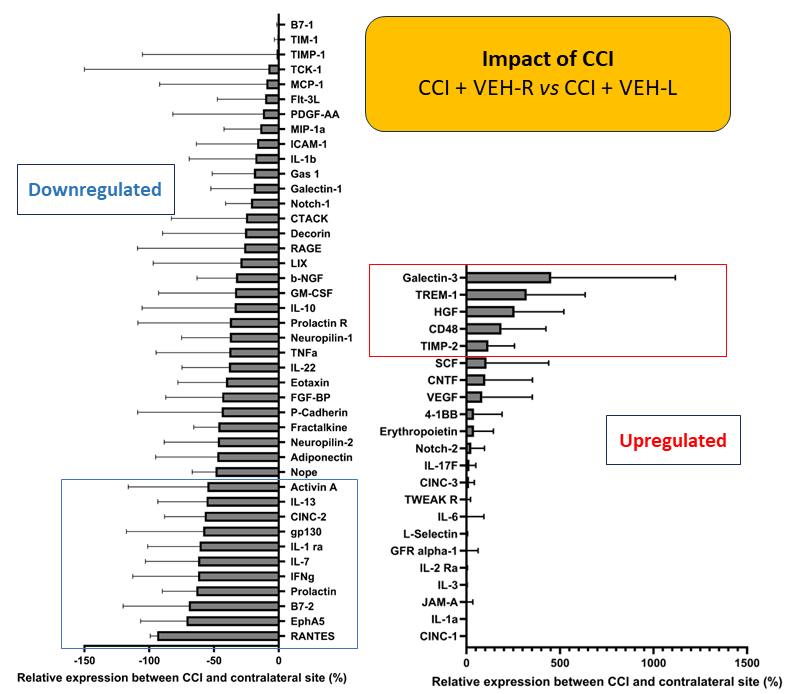


**Figure S4.** **Tissue cytokine ELISA assay showing the impact of CCI on cytokine expression.** The cytokine ELISA assays were performed in quadruplicates to analyze differences between treatment groups and hemispheres using tissue lysates (prepared as shown in Fig. S7). Bar graphs (mean and standard deviation) comparing the TBI and contralateral stes of untreated rats showing the impact of TBI on cytokine expression levels.


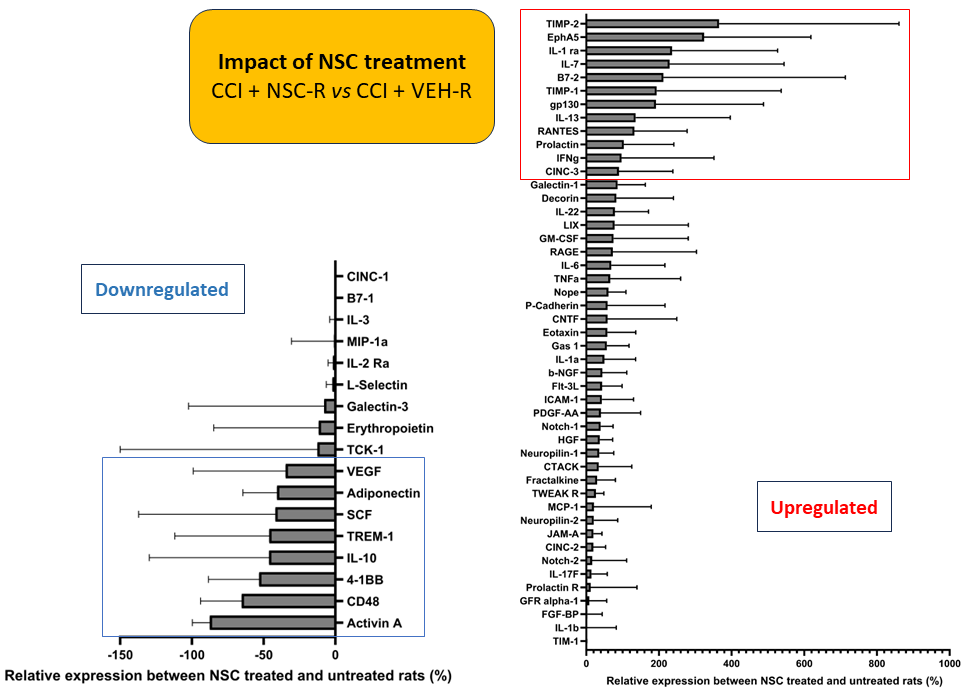


**Figure S5.** **Tissue cytokine ELISA assay showing the impact of NSC treatment on cytokine expression.** The cytokine ELISA assays were performed in quadruplicates to analyze differences between treatment groups and hemispheres using tissue lysates (prepared as shown in Fig. S7). Bar graphs (mean and standard deviation) comparing cytokine expression profiles of treated rats (LMNSC008) to untreated rats (vehicle) showing the impact of NSC administration on cytokine expression levels of animals that had received a CCI.


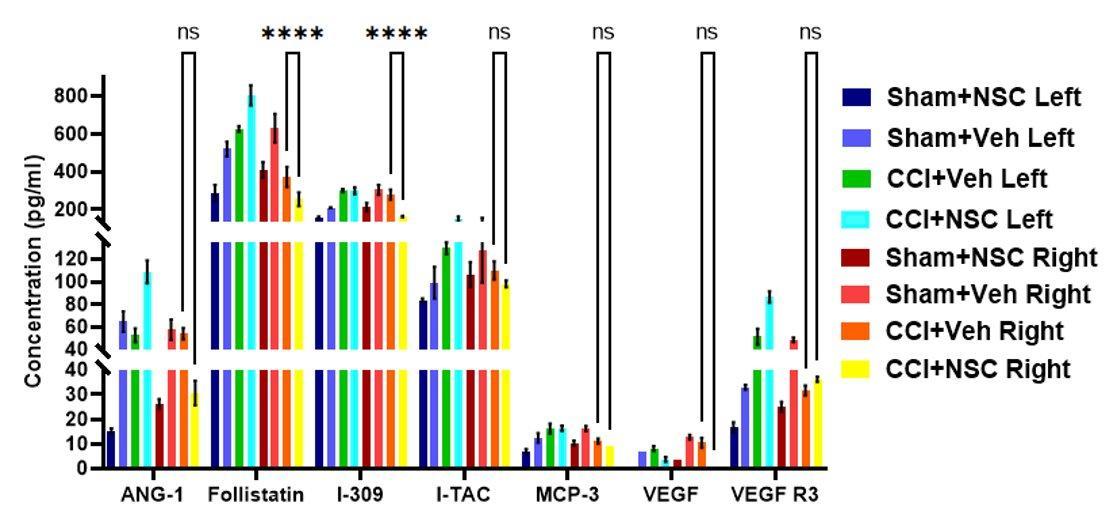


**Figure S6. Tissue angiogenesis ELISA assay.** Results obtained from female rat brain lysates of contralateral (left hemisphere, blue-scale) and CCI (right hemisphere, red-scale) samples. Lysates were prepared from manually excised sections of the left and right hemispheres (as shown on Fig. S7) aligned with the CCI damage site through the hippocampus on rat brains from the four treatment groups left and right (SHAM + NSC, SHAM + VEH, CCI + VEH, and CCI + NSC). The angiogenesis ELISA assay (QAH-ANG-3, RayBiotech) was performed in quadruplicates to analyze differences between treatment groups and hemispheres for proteins ANG-1, Follistatin, I-309, I-TAC, MCP-3, VEGF, and VEGF-R3. Graphs constructed with data and analysis from a PMT500 scan with a GenePix Microarray Scanner (4100A). Asterisks denote statistically significant difference between CCI + VEH and CCI + NSC treatment groups in the right hemisphere (P-value <0.0001). Error bars represent the standard deviation.

**Figure S7.** Schematic diagram of tissue dissection and preparation for ELISA and NanoString analysis.

 **Figure S8.** **IHC staining of rat brain** using (A) human nestin specific antibodies: a1 inset; (B) Multiplex IHC image, staining TBI injury site after treatment with LMNSC008 cells IBA1-yellow, CD31-purple, CD68-teal.


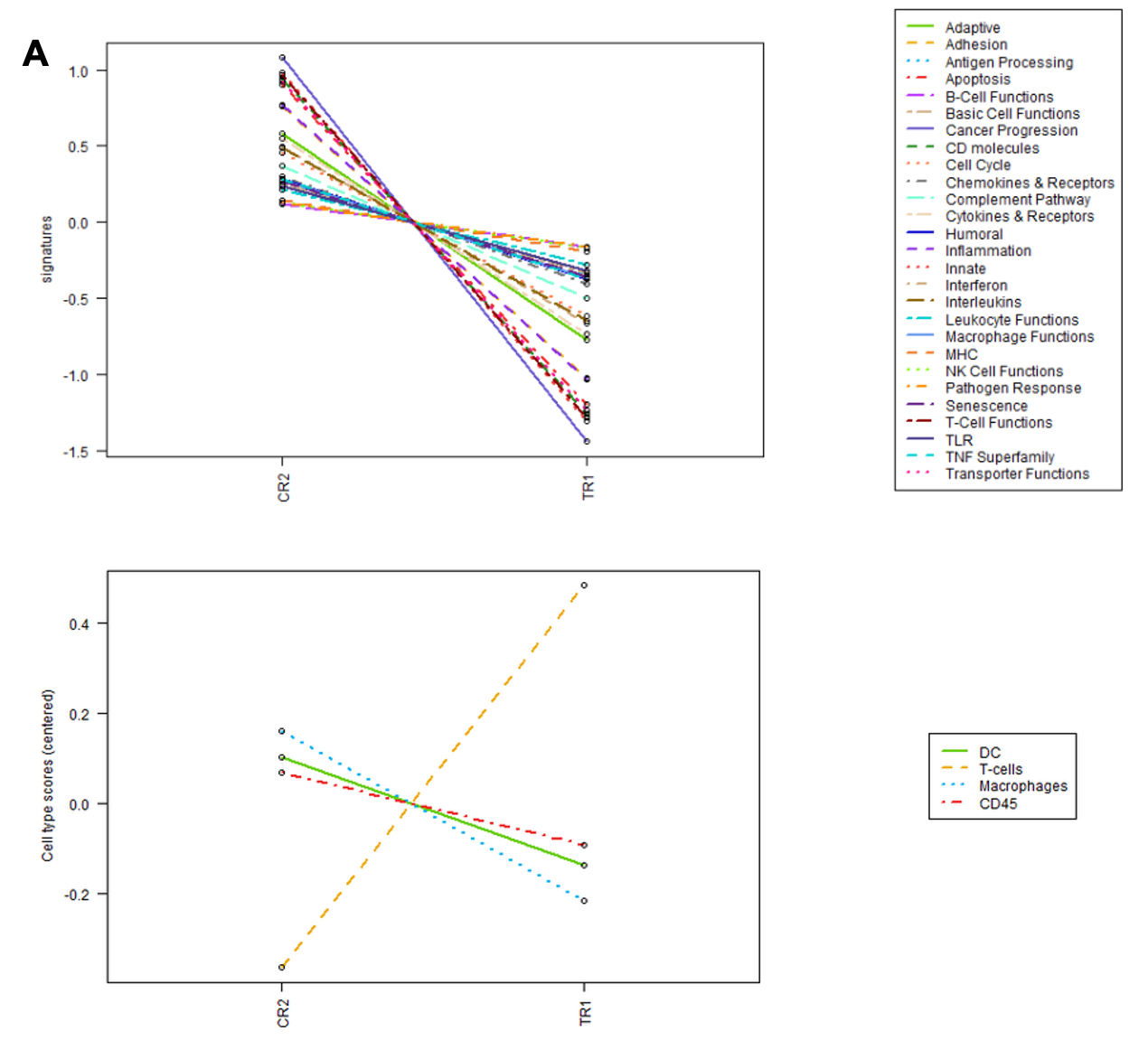


**Figure S9.** (A) Pathway score summary when CR2 (CCI+ VEH) and TR1 (CCI + NSC) groups were analyzed using NanoString data. Please see table S1 for the sample attribute list used for NanoString analysis. (B) Cell Type differential analysis of immune cell types.


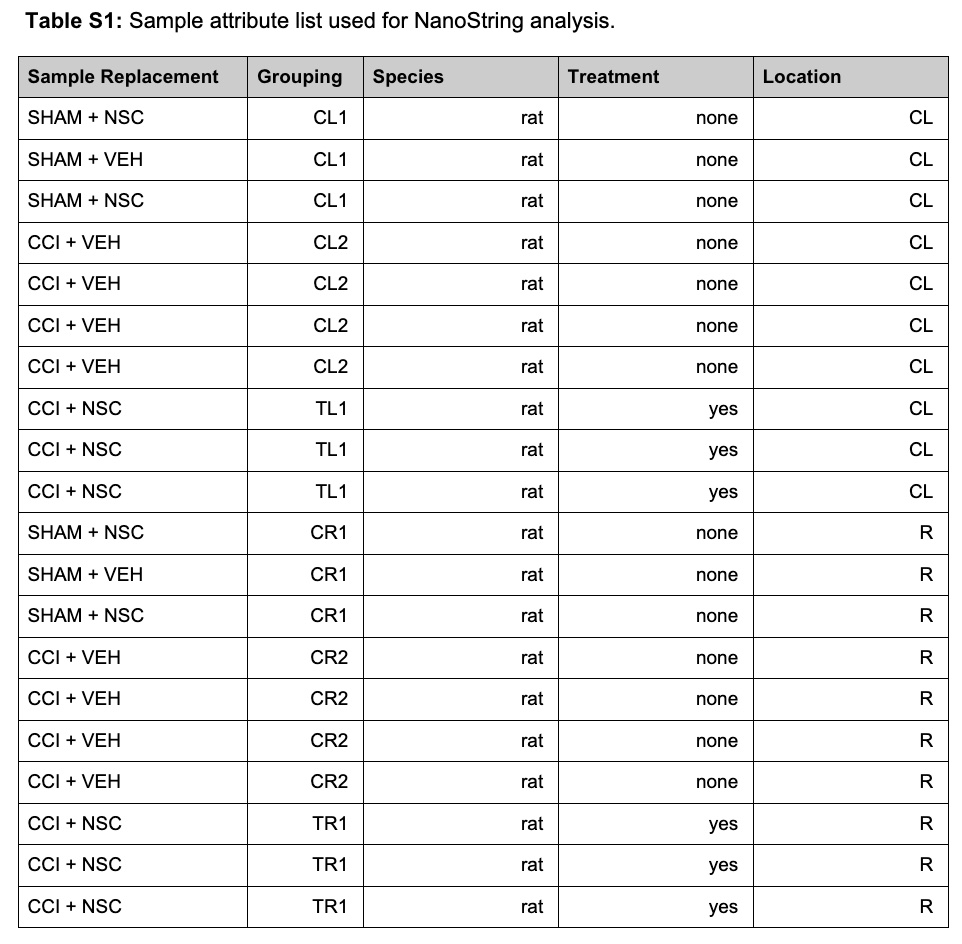


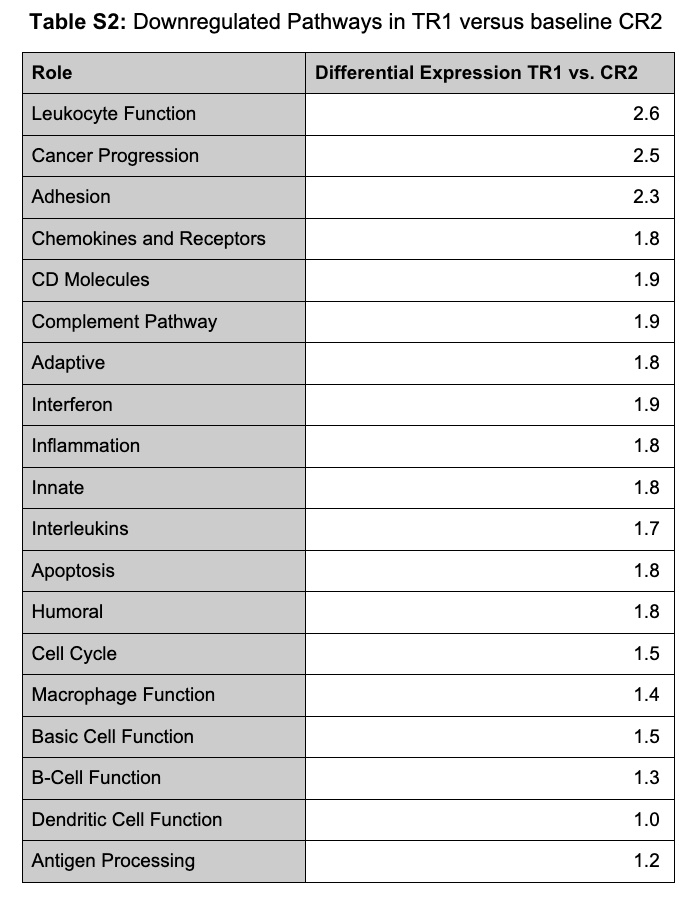

Supplement: Supplementary file 1 — Supplementary Information. [file 41598_2023_44426_MOESM1_ESM.docx]
